# sEMG-Based Motion Intention Recognition for Interactive Upper Limb Nursing Assistance

**DOI:** 10.3390/s26103021

**Published:** 2026-05-11

**Authors:** Zekun Peng, Yongfei Feng, Liangda Wu, Jiaxing Cheng, Xiaohui Fang

**Affiliations:** 1 School of Mechanical Engineering, Shanghai Jiao Tong University, Shanghai 200240, China; 2 Faculty of Mechanical Engineering & Mechanics, Ningbo University, Ningbo 315211, China; 3 School of Mechanical Engineering, Yangzhou University, Yangzhou 225009, China; 4 Yangzhou Command AI Technology Co., Ltd., Yangzhou 225127, China

**Keywords:** surface electromyography, motion intention recognition, interactive nursing assistance, feature fusion

## Abstract

**Highlights:**

**What are the main findings?**

- A structured sEMG-based framework integrating acquisition, feature extraction, and classification enables reliable motion intention recognition for upper-limb assistance.
- A compact time-domain feature set (RMS, IF, CF) combined with an optimized Random Forest could achieve high accuracy in both offline and real-time validation.

**What are the implications of the main findings?**

- The proposed lightweight feature–model combination supports real-time implementation with low computational cost for assistive and rehabilitation systems
- The system-level integration of sEMG decoding with deterministic control can improve stability and interpretability in human–robot interaction.

**Abstract:**

Surface electromyography (sEMG) enables non-invasive acquisition of neuromuscular activity and has shown strong potential for motion intention recognition in human–machine interaction. However, achieving reliable and real-time decoding remains critical for interactive upper-limb assistance. This study presents a structured sEMG-based framework for motion intention recognition in upper-limb assistance tasks, integrating multi-channel acquisition, standardized preprocessing, time-domain feature extraction, and supervised learning. sEMG signals from four representative motions were collected, and eight time-domain features were extracted from denoised and segmented signal windows. A compact feature subset was identified through systematic evaluation. Five classifiers were benchmarked under consistent validation conditions, with Random Forest achieving the best performance and further optimized via K-fold cross-validation. The proposed method achieved an average intra-subject accuracy of 95.23% across eight subjects and 95.72% in online interactive validation. These results demonstrate that time-domain feature fusion combined with ensemble learning provides robust and efficient motion discrimination, highlighting its potential for real-time assistive and rehabilitation applications.

## 1. Introduction

With the rapid growth of aging populations and the increasing demand for long-term daily care, interactive upper-limb assistive technologies are becoming an essential component of intelligent nursing systems [1,2,3,4]. Many routine assistance tasks—such as guided arm elevation, cup-to-mouth lifting, stable holding, or repetitive flexion–extension—require precise synchronization between a user’s voluntary intention and the mechanical response of the device [5]. In such human–machine interaction scenarios, the system must infer user intent with minimal latency and execute assistance actions in a predictable and stable manner [6]. Conventional intention inference methods based on kinematic sensing or force/torque measurements typically respond only after observable limb displacement or physical interaction forces have occurred. This reactive nature introduces temporal delay, limits anticipatory control, and may lead to unintended activation when voluntary movement is subtle or incomplete [7,8]. Therefore, physiologically grounded intention decoding strategies are increasingly regarded as a key enabler for responsive and reliable interactive assistance.

sEMG offers a direct window into neuromuscular activation associated with voluntary motor commands. Because muscle activation precedes large joint motion, sEMG-based decoding has the potential to reduce response latency and improve interaction smoothness compared with purely mechanical sensing [9,10,11]. However, the practical implementation of sEMG-driven intention recognition remains challenging. sEMG signals exhibit low amplitude, stochastic variability, and high sensitivity to motion artifacts, electrode displacement, and environmental interference. Moreover, multi-muscle activation patterns often overlap across different functional movements, especially during movement onset and transition phases, reducing inter-class separability [12,13]. When such recognition modules are embedded within real-time interaction loops, even minor instability or transient misclassification can propagate into inappropriate mechanical responses. Consequently, robust sEMG-based intention recognition requires not only accurate classification algorithms but also stable acquisition protocols and reliable execution constraints. For clarity, key terms used in this study are defined as follows. Motion intention refers to the voluntary neuromuscular activation associated with a user’s intended movement prior to observable limb motion. Interactive nursing assistance denotes a closed-loop human–robot interaction process in which the assistive device responds to inferred user intent in real time. Rehabilitation applications refer to assistive or training scenarios aimed at restoring or supporting motor function through guided or intention-driven movements.

Extensive research has investigated sEMG preprocessing, feature extraction, and classification modeling. Feature representations are typically categorized into time-domain, frequency-domain, and time–frequency descriptors [14,15]. While spectral and time–frequency approaches can capture additional signal characteristics, they often introduce transformation overhead and parameter sensitivity that may limit real-time deployment. Time-domain features, by contrast, can be computed directly from short sliding windows with low latency, making them attractive for interactive systems [16,17,18,19,20]. On the modeling side, linear discriminant methods provide computational efficiency but may underperform when the feature space is not linearly separable. Nonlinear classifiers—including kernel-based models and ensemble learning methods—have demonstrated improved discrimination in complex multi-channel sEMG scenarios. However, reported performance often depends strongly on acquisition consistency, feature redundancy, and evaluation protocols, particularly when transitioning from offline analysis to online interaction contexts. Nevertheless, unlike existing studies that primarily focus on improving classification accuracy using increasingly complex models, this work emphasizes a system-level design that enables real-time, interpretable, and deployable sEMG-based intention recognition for interactive assistive applications [21].

Despite substantial methodological advances, two practical limitations remain particularly relevant for interactive upper-limb assistance systems. First, many studies emphasize offline classification accuracy without embedding the recognition pipeline into a clearly structured interaction architecture that integrates standardized motion prompting, synchronized multi-channel acquisition, and deterministic command execution. As a result, the transition from laboratory evaluation to real-time operation is often insufficiently examined [22,23]. Second, feature sets are frequently expanded in pursuit of marginal accuracy gains, leading to increased dimensionality and reduced interpretability when the dataset size is moderate. For closed-loop assistive interaction, recognition performance must be evaluated under a reproducible acquisition protocol, a compact and discriminative feature representation, and an execution interface that explicitly constrains unintended activation [24,25,26]. Without such structural coherence, high offline accuracy does not necessarily translate into stable online behavior.

To address these issues, this study proposes a structured sEMG-based motion intention recognition framework for interactive upper-limb nursing assistance. The system is implemented on a hardware–software integrated platform under controlled laboratory conditions. The mechanical assistance platform employed in this study is derived from a previously reported variable-height rotating end-traction upper limb robotic system [27,28], which provides the physical basis for the interactive nursing assistance considered here. In the present work, an sEMG-driven intention recognition module is integrated into this platform to enable intention-based rather than purely pre-programmed assistance. The workflow is organized into offline model construction and online execution stages. Time-domain features are extracted from segmented sEMG windows and evaluated under a unified protocol, followed by comparative assessment of multiple supervised classifiers. The selected model is embedded into the online interaction loop, where predicted motion labels are deterministically mapped to control commands with a temporal consistency constraint to enhance execution stability.

Although several components used in sEMG-based motion recognition have been widely studied, practical deployment in interactive assistance systems requires a structured framework that integrates physiological signal decoding with deterministic device control. In this work, the novelty lies primarily in the system-level integration and experimental validation of an sEMG-driven interaction pipeline, rather than in proposing a new standalone classification algorithm. The main contributions of this study are summarized as follows:

(1) Unlike many existing studies that focus on offline recognition performance, this work establishes a complete pipeline from signal acquisition, preprocessing, feature fusion, and classification to real-time execution in an interactive assistive system. The framework is designed with low-latency constraints, enabling practical deployment in closed-loop human–robot interaction scenarios.
(2) Instead of relying on computationally intensive deep learning models, the proposed approach adopts a compact feature set (RMS, IF, CF) combined with an optimized Random Forest classifier, achieving a balance between accuracy, computational efficiency, and interpretability, which is critical for real-time rehabilitation and assistive applications.
(3) In addition to offline evaluation, the framework is validated through online interactive experiments, demonstrating stable performance under real-time conditions. This bridges the gap between conventional sEMG-based recognition studies and practical assistive system implementation.

## 2. Materials and Methods

### 2.1. System Framework

**Figure 1:**
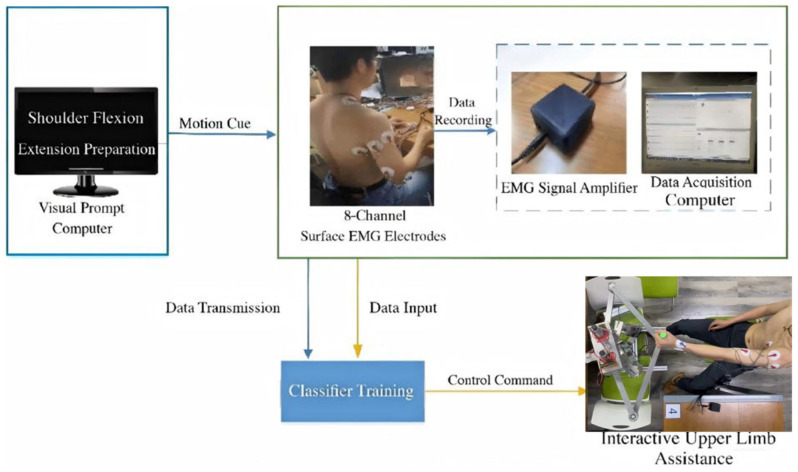
Experimental framework of the sEMG-driven interactive upper limb assistance system.

The experimental framework for sEMG-driven interactive upper limb assistance is illustrated in Figure 1. The system is organized as a data-driven closed-loop interaction process that integrates motion prompting, multi-channel sEMG acquisition (EMGduino amplifier, developed by Hangyi Bio-tech Co., Ltd., Hangzhou, China), model training, and assistance execution. Unlike purely simulation-based architectures, the proposed setup reflects a real hardware–software implementation under controlled laboratory conditions. The interaction process begins with a motion cue displayed on Computer 1, which provides standardized visual prompts to guide the participant in performing predefined upper limb actions. These cues ensure consistent timing and reduce variability during data acquisition. Upon receiving the motion instruction, the participant executes the corresponding action while muscle activity is recorded.

Surface EMG signals are captured using a standard multi-channel bipolar measurement setup with wet electrodes placed over selected upper limb muscles. The acquired analog signals are amplified by an sEMG signal amplifier before being transmitted to Computer 2 for digital recording. The recorded signals consisted of synchronized multi-channel sEMG data, with each channel corresponding to a specific muscle site. All channels were acquired simultaneously and stored for subsequent processing. Data flow within the system consists of two distinct stages, offline model training and online assistance execution. During the training phase, recorded sEMG data are input into the processing pipeline for feature extraction and classifier construction. The trained model maps feature vectors to motion categories. Once the model is established, predicted motion labels are converted into control commands through a deterministic mapping function. Such separation enhances interpretability and allows independent evaluation of the sEMG recognition framework without confounding factors introduced by adaptive control algorithms.

To ensure reliable intention recognition, representative upper limb actions were predefined and presented through visual prompts. The motion set is defined as

***Y*** = {*y*_1_,*y*_2_,*y*_3_,*y*_4_} (1)

corresponding to a static holding posture, a composite lifting motion simulating drinking assistance, forearm flexion–extension and shoulder abduction. Each motion trial follows a standardized timing structure consisting of preparation, execution, and return phases. The preparation cue appears on Computer 1, followed by the instructed movement. Participants were instructed to maintain a comfortable execution speed and consistent movement amplitude across repetitions.

Motion labels predicted during the classification stage are converted into executable control commands for the upper limb assistance platform. For each segmented sEMG window, the classifier produces a motion category, which is subsequently mapped to a predefined control signal through a deterministic command mapping function. This mapping is implemented as a discrete command selection mechanism rather than a continuous proportional control scheme. The generated control signal is transmitted to the assistance device to trigger the corresponding movement. To ensure operational stability, command updates are synchronized with the segmentation interval defined in the sEMG processing stage. Assistance actions are executed only when classification outputs remain consistent across consecutive windows, thereby reducing unintended activation caused by transient signal fluctuations or short-term misclassification. This temporal consistency constraint enhances interaction reliability without introducing additional control-layer complexity.

The complete operational workflow is physiologically driven and follows a sequential process beginning with motion prompting and voluntary neuromuscular activation. The resulting sEMG signals are recorded, processed into structured feature representations, and classified into motion categories. The predicted category is then translated into a control command that drives the assistance platform. By maintaining a clear distinction between signal decoding and mechanical execution, the framework enables systematic evaluation of the sEMG-based intention recognition module under realistic interaction conditions.

### 2.2. sEMG Acquisition and Experimental Design

#### 2.2.1. sEMG Acquisition Configuration

Surface electromyography was employed as the physiological interface for motion intention recognition in interactive upper limb assistance. The surface signal represents the superposition of motor unit action potentials activated by descending neural commands. This property makes sEMG suitable for intention-driven human–machine interaction, where timely decoding of user intent is essential for responsive assistance. However, the signal amplitude is typically low and sensitive to motion artifacts, electrode displacement, and environmental interference. Therefore, acquisition procedures were standardized to ensure signal stability and reproducibility.

A multi-channel sEMG acquisition system was used to record muscle activity from selected upper limb muscles. Let the acquired signal be defined as

***x***(*t*) = [*x*_1_(*t*),*x*_2_(*t*),*x_m_*(*t*),…,*x*_8_(*t*)]^T^ (2)

where *m* denotes the number of sEMG channels and *x*_m_(*t*) represents the time-varying voltage recorded from the *m*-th electrode pair. All channels were synchronously sampled at a fixed sampling frequency to preserve temporal alignment across muscles.

**Figure 2:**
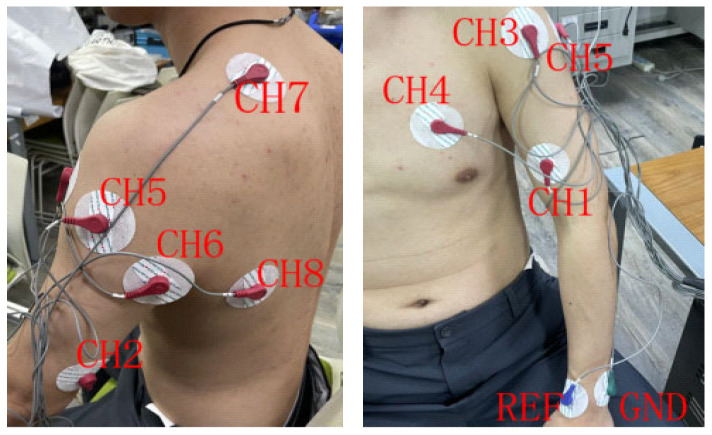
Electrode placement and multi-channel sEMG acquisition setup.

An eight-channel bipolar sEMG acquisition setup was employed to capture upper-limb muscle activity. Electrodes were placed over eight representative muscles, including the biceps brachii, triceps brachii, anterior deltoid, pectoralis major, lateral deltoid, posterior deltoid, trapezius, and latissimus dorsi. A reference electrode was attached to a neutral bony region, and a ground electrode was used to reduce common-mode noise. Electrode placement, as illustrated in Figure 2, followed a standard bipolar configuration aligned with the muscle fiber orientation to reduce cross-talk and improve spatial selectivity. Prior to attachment, the skin surface was cleaned to minimize impedance variability. After electrode placement, a short verification recording was conducted to confirm waveform stability and to exclude saturation or excessive baseline drift. During acquisition, real-time waveform visualization was monitored to detect abnormal fluctuations. To minimize environmental interference, all recordings were conducted in a controlled laboratory setting. Participants were seated in a stable posture to reduce non-task-related body motion and limit motion artifacts unrelated to the target upper-limb movements.

Eight healthy adult participants (university students, age: 21–26 years) were recruited for this study. All participants had no known neuromuscular or musculoskeletal disorders and were able to perform the required upper-limb movements. Individuals with a history of upper-limb injury or neurological impairment were excluded. Written informed consent was obtained from all participants prior to the experiment.

#### 2.2.2. Experimental Protocol and Motion Organization

**Figure 3:**
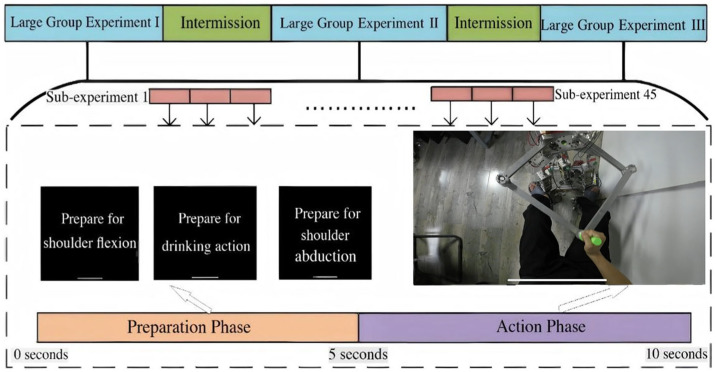
Experimental block structure and timing of motion trials.

To construct a reliable dataset for supervised intention recognition, a structured experimental protocol was developed around representative upper limb assistance tasks as shown in Figure 3. The purpose of this design was to capture distinguishable neuromuscular activation patterns associated with voluntary motion intentions under controlled conditions. The experiment was organized into multiple blocks separated by rest intervals to mitigate fatigue-induced signal drift. Each block contained several sub-trials corresponding to predefined motion categories. Each category represents a functional upper limb action relevant to assistance scenarios.

Within each sub-trial, a unified temporal structure was adopted, consisting of preparation and execution phases. A visual cue presented the instructed motion, after which the participant executed the movement at a comfortable self-selected speed. The preparation phase ensured a consistent baseline state, while the execution phase captured voluntary muscle activation corresponding to the intended action.

Let the total duration of a sub-trial be denoted as *T*, and the effective contraction interval as *T_eff_* ⊂ *T.* Only the execution segment within *T_eff_* was retained for subsequent analysis, as transitional segments may contain mixed activation patterns that introduce ambiguity into class labels. Rest intervals were incorporated between blocks to reduce muscle fatigue and preserve signal stationarity. By maintaining consistent movement amplitude and posture constraints across repetitions, the protocol balanced ecological validity with experimental control.

#### 2.2.3. Data Segmentation and Window Modeling

Following the acquisition, the continuous multi-channel sEMG stream was segmented according to synchronized temporal markers recorded during the experiment. Although each sub-trial contained preparation and return phases, only the steady contraction interval during the execution phase was retained for analysis. Transitional segments often include gradual activation onset, deceleration, or posture adjustment, which may produce mixed neuromuscular patterns and reduce class separability. Therefore, excluding these intervals ensured that retained segments corresponded to relatively stable voluntary activation states.

Let the execution interval of the *i*-th sub-trial be denoted as [*t_i_*_,*s*_,*t_i_*_,*e*_], where *t_i_*_,*s*_ and *t_i_*_,*e*_ represent the onset and offset timestamps of steady contraction. The retained signal segment for this sub-trial can be written as ***x****_i_*(*t*), *t* ∈ [*t_i_*_,*s*_,*t_i_*_,*e*_]. To enable structured feature extraction and consistent model training, each retained segment was further divided into fixed-length analysis windows. Assuming a sampling frequency *f_s_* and window duration Δ*t*, the number of samples per window is

*N* = *f_s_*∙Δ*t* (3)

A windowed signal segment is therefore defined as

***X****_k_* = {***x***(*t_k_*),***x***(*t_k_* + 1),…,***x***(*t_k_* + *N* − 1)} (4)

where *k* indexes the starting sample of the window.

Windowing was performed using a constant window length across all motion categories to maintain uniform temporal resolution. If overlapping windows were used, the stride between consecutive windows was kept fixed to preserve structural consistency in the dataset. This sliding-window representation transforms the continuous sEMG stream into a sequence of quasi-stationary segments suitable for subsequent statistical characterization. The choice of fixed-length windowing is consistent with common sEMG pattern recognition practice, where short-duration segments approximate local stationarity of the stochastic signal. By applying identical segmentation parameters across all sub-trials, the resulting dataset maintains comparability across motion classes and prevents bias introduced by unequal temporal sampling density.

### 2.3. Signal Processing and Feature Extraction

#### 2.3.1. sEMG Signal Preprocessing

Raw sEMG signals were subjected to standardized preprocessing prior to feature extraction to enhance signal quality and suppress noise contamination. Due to the low amplitude and stochastic characteristics of sEMG, the recorded signals may contain baseline drift, high-frequency noise, and periodic electrical interference introduced by acquisition hardware and environmental factors. Therefore, a multi-stage preprocessing pipeline was applied uniformly across all channels. An initial channel quality inspection was conducted to identify abnormal channels exhibiting excessive baseline offset, saturation, or unstable amplitude fluctuations. Segments affected by electrode detachment or transient artifacts were excluded to prevent distortion of subsequent feature computation. Minor abnormal offsets were corrected using local interpolation to preserve temporal continuity. This preliminary inspection ensured that downstream processing operated on structurally consistent data.

During acquisition, sEMG signals were sampled at 500 Hz using a multi-channel sEMG acquisition system. After removing unstable segments, the signals were segmented using a sliding-window strategy for feature extraction. The window length was set to 200 ms, corresponding to 100 samples per window, and the step size was 100 ms, resulting in a 50% overlap between adjacent windows. This configuration provides a balance between temporal resolution and feature stability for sEMG pattern recognition.

To suppress stochastic noise while preserving transient muscle activation characteristics, wavelet-based denoising was employed. Multi-level wavelet decomposition using the Daubechies-4 (db4) mother wavelet was applied with a four-level decomposition. Wavelet coefficients were processed using a soft-thresholding rule to attenuate high-frequency noise components before signal reconstruction.

After wavelet denoising, a band-pass filter ranging from 20 Hz to 450 Hz was applied to retain the dominant physiological frequency band of surface sEMG signals. In addition, a 50 Hz notch filter was used to suppress power-line interference. All preprocessing parameters were kept consistent across subjects and experimental sessions. The wavelet denoising process can be mathematically expressed as

*x_w_*(*t*) = *W*^−1^(*T*(*W*(*x*(*t*)))) (5)

where *W*(·) denotes the wavelet transform, *T*(·) the threshold operation applied to wavelet coefficients, and *W*^−1^(·) the inverse transform. This procedure attenuates high-frequency noise while maintaining signal morphology.

The filtering operation can be described as

*x_f_*(*t*) = *x_w_*(*t*) ∗ *h*(*t*) (6)

where *h*(*t*) represents the impulse response of the band-pass filter and ∗ denotes convolution. Filtering parameters were kept constant across all recordings to ensure consistency in spectral characteristics. After the combined denoising and filtering steps, the processed sEMG signals exhibited improved amplitude stability and reduced interference, providing a reliable basis for subsequent feature extraction.

#### 2.3.2. Multi-Feature Fusion Strategy

Surface EMG feature extraction methods can generally be categorized into time-domain, frequency-domain, and time–frequency approaches. In this study, time-domain features were adopted to maintain computational efficiency and real-time compatibility. Unlike spectral-based representations that require additional transformation steps, time-domain descriptors can be directly computed from segmented sEMG windows, making them suitable for online motion intention recognition systems.

Eight time-domain features were extracted from each segmented sEMG window. These descriptors capture amplitude characteristics, distribution properties, and peak-related behavior of the signal. Mean Absolute Value (MAV) represents the average absolute amplitude of the signal within a window and reflects the overall deviation of sEMG activity from the baseline. Variance (VAR) measures the dispersion of the signal around its mean value and characterizes amplitude fluctuation intensity. Root Mean Square (RMS) quantifies the effective signal power and is widely used to indicate muscle contraction strength. Waveform Factor (WF) is defined as the ratio between RMS and MAV and describes waveform distortion characteristics in the time domain. Skewness (SK) evaluates the asymmetry of the signal distribution relative to its mean. Kurtosis (KU) measures the peakedness of the distribution and is sensitive to abrupt amplitude variations. Impulse Factor (IF) is the ratio between peak amplitude and mean absolute value and emphasizes extreme signal behavior. Clearance Factor (CF) is defined as the ratio between peak amplitude and RMS value and is commonly used to characterize peak prominence. These eight descriptors form the candidate feature pool for subsequent performance evaluation and feature selection.

To assess the discriminative capability of individual features, each feature was independently used to construct a feature vector for classification using a single classifier under identical validation conditions. Classification accuracy served as the evaluation metric. To prevent data leakage, the dataset was partitioned at the trial level prior to segmentation, and all model selection procedures were performed exclusively on the training data. The performance results are summarized in Table 1. RMS achieved the highest average performance among single features, followed by IF and CF. VAR exhibited comparatively weaker performance, indicating limited discriminative contribution under the current experimental configuration.

Although RMS demonstrated superior standalone performance, single-feature representation was insufficient to fully characterize inter-class differences. Therefore, feature fusion was conducted by combining high-performing descriptors. Candidate combinations were constructed by progressively integrating top-performing features identified in single-feature evaluation. Each combination was evaluated using the same classifier and validation protocol. The results are presented in Table 2.

The combination of IF, CF, and RMS achieved the highest overall accuracy. Adding additional features beyond this triplet did not yield further improvement and, in some cases, slightly reduced performance, suggesting potential redundancy or over-parameterization. Based on these evaluations, the triplet feature set consisting of RMS, IF, and CF was selected as the final representation for subsequent classifier construction.

## 3. Results

### 3.1. Experimental Setup and Evaluation Protocol

The classification experiments were conducted using the fused time-domain feature set (RMS, IF, and CF). Data were collected from eight subjects performing four upper limb motion categories: seated rest, drinking, forearm flexion–extension, and shoulder abduction. For each subject, the recorded motion trials were first divided into training and testing subsets. Specifically, 90% of the motion trials were assigned to the training set, while the remaining 10% were reserved for testing. To avoid potential information leakage, the partitioning was performed at the trial level before the sliding-window segmentation step. After this separation, signal segmentation and feature extraction were applied independently within the training and testing subsets. Consequently, sEMG windows derived from the same motion trial were not shared between the two datasets, ensuring that temporal correlations within trials did not artificially inflate classification accuracy. This strategy was applied independently to each subject to preserve intra-subject consistency. The same data splitting protocol was maintained across all classifiers to ensure comparability. It should be noted that the current evaluation focuses on intra-subject performance; cross-subject generalization was not assessed in this study and will be addressed in future work.

All classification algorithms were implemented in Python 3.6. The Random Forest and Gradient Boosting models were constructed using the scikit-learn library, while SVM, LDA, and QDA were implemented using their corresponding standard modules. Classification performance was evaluated using recognition accuracy, defined as the proportion of correctly classified samples over the total number of test samples:

*Accuracy* = *N*_correct_/*N*_total_ (7)

where *N*_correct_ denotes the number of correctly predicted samples and *N*_total_ represents the total number of samples in the test set.

To provide a consistent evaluation framework, all classifiers were trained and tested under identical conditions, including feature representation, data partitioning, and evaluation metric.

### 3.2. Comparative Evaluation of Classification Models

**Figure 4:**
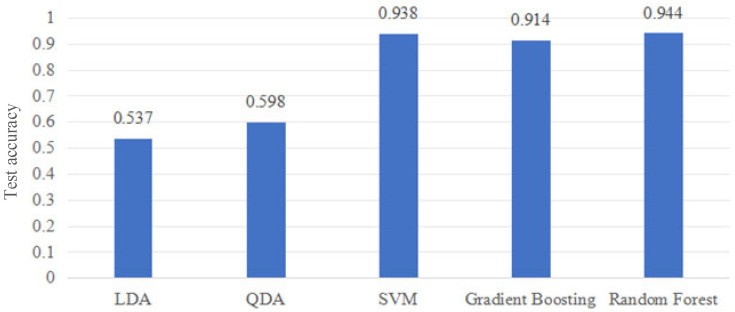
Average test accuracy of different classifiers across eight subjects.

To evaluate the effectiveness of different classification strategies for sEMG-based motion intention recognition, five supervised learning algorithms were compared under the unified experimental protocol. These included Support Vector Machine (SVM, RBF kernel), Linear Discriminant Analysis (LDA), Quadratic Discriminant Analysis (QDA), Gradient Boosting (GB), and Random Forest (RF). All classifiers were trained and tested independently for each subject using the same feature representation and 90–10% train–test split. As shown in Figure 4, significant differences in recognition performance were observed among classifiers.

Linear Discriminant Analysis yielded the lowest overall accuracy (0.537), indicating insufficient linear separability of the extracted sEMG feature space. Although Quadratic Discriminant Analysis slightly improved performance (0.598), the accuracy remained limited, suggesting that class covariance modeling alone was insufficient to capture inter-class variability. In contrast, nonlinear classifiers demonstrated substantially higher recognition accuracy. SVM with RBF kernel achieved an average accuracy of 0.938, confirming the effectiveness of kernel-based nonlinear mapping for sEMG motion classification. Gradient Boosting also provided stable performance (0.914), benefiting from its ensemble structure. Among all evaluated models, Random Forest achieved the highest average test accuracy (0.944). The performance difference between Random Forest and SVM was modest (0.006), but Random Forest consistently produced slightly higher recognition rates across subjects.

In addition to overall accuracy, per-class performance under Random Forest was analyzed. The average class-wise recognition rates across subjects were 0.952 (seated rest, *y*_1_), 0.941 (drinking, *y*_2_), 0.943 (forearm flexion–extension, *y*_3_), and 0.943 (shoulder abduction, *y*_4_). The relatively balanced distribution indicates that the classifier does not exhibit significant class bias under the current feature representation. Ensemble-based classifiers demonstrated superior performance compared to linear discriminant methods. These findings suggest that nonlinear decision boundaries are better suited to characterize the discriminative structure of the selected sEMG time-domain features.

### 3.3. Hyperparameter Optimization of the Selected Random Forest Model

Among the evaluated classifiers, Random Forest achieved the highest average recognition accuracy (0.944) across eight subjects, slightly outperforming SVM (0.938) and Gradient Boosting (0.914). In addition to its superior accuracy, Random Forest demonstrated stable class-wise performance under identical experimental conditions. Therefore, Random Forest was selected as the primary model for further optimization.

**Figure 5:**
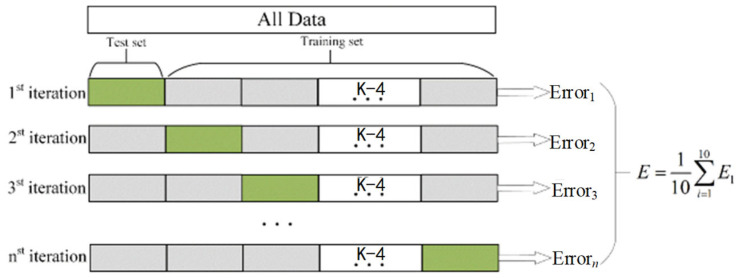
Schematic illustration of the K-fold cross-validation procedure.

Although the baseline Random Forest already provided competitive performance, its predictive capability depends on appropriate hyperparameter selection. To further enhance generalization performance, hyperparameter tuning was conducted using K-fold cross-validation as shown in Figure 5. The dataset of each subject was partitioned into K mutually exclusive subsets of equal size. In each iteration, K − 1 subsets were used for training, and the remaining subset was used for validation. The process was repeated K times, and the average validation accuracy was used to evaluate model performance.

To further improve model performance, a grid-based hyperparameter search was conducted using K-fold cross-validation. Two key parameters of the Random Forest model were optimized: the number of decision trees (n_estimators) and the maximum depth of each tree (max_depth). For each parameter combination, cross-validation was performed to estimate generalization performance under identical data partitioning conditions. The results indicated that model performance stabilized as the number of trees increased, while excessively shallow tree depth limited discriminative capacity. The optimal configuration was obtained when n_estimators was set to 181 and max_depth to 131. This parameter combination yielded the highest cross-validation accuracy and was therefore adopted for subsequent evaluation.

Using the optimized parameters, the classification experiments were repeated for all eight subjects under the same evaluation protocol. After optimization, the Random Forest model achieved an average recognition accuracy of 0.9523 across subjects as shown in Table 3. Compared with the baseline performance (0.944), this corresponds to an improvement of 0.0083. Although the numerical increase appears modest, it confirms that systematic hyperparameter tuning contributes to measurable performance enhancement under the current dataset scale.

### 3.4. Online Validation Experiment

To evaluate the real-time feasibility of the proposed sEMG-based motion intention recognition framework, an online validation experiment was conducted using an independent subject under interactive system conditions. The experiment was designed as a preliminary proof-of-concept test to verify whether the trained model could operate under real-time interactive conditions.

Prior to the experiment, a healthy participant was recruited. The participant was instructed about the experimental procedure and allowed to rest adequately before data collection. During the experiment, motion cues were presented on the control interface, and the participant voluntarily executed the corresponding upper limb movements. Five motion groups were first collected to train the model. After model training was completed, three testing rounds were conducted. Each round included four motion categories: seated rest, forearm flexion–extension, drinking, and shoulder abduction. Each motion was repeated 15 times. A single motion cycle lasted 5 s, followed by a 5 s return and rest period. The total duration per round was approximately 450 s. Extracted fused features (RMS, impulse factor, and clearance factor) were fed into the trained Random Forest classifier for real-time prediction.

**Figure 6:**
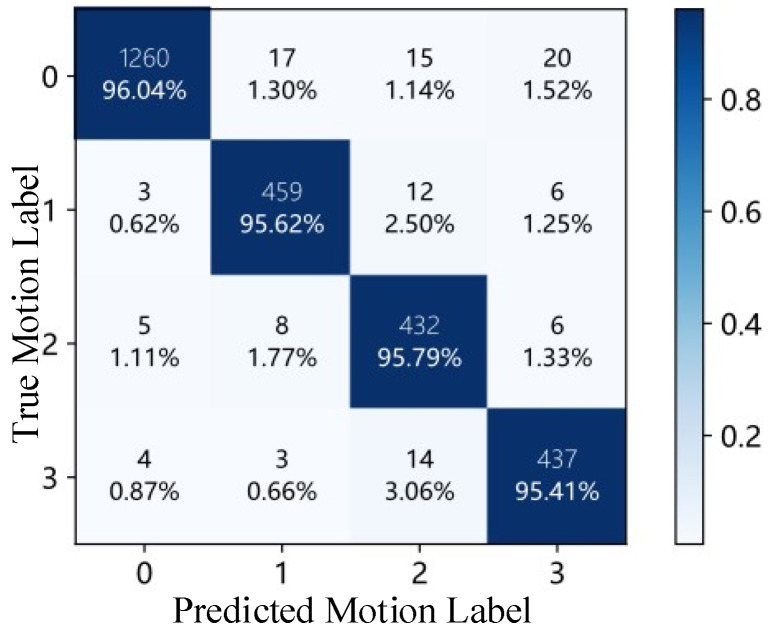
Confusion matrix of the optimized Random Forest classifier in the online validation experiment.

The confusion matrix of the online validation experiment is shown in Figure 6. The horizontal axis represents predicted labels, and the vertical axis represents true motion labels. The diagonal entries correspond to correctly classified samples. The overall average recognition accuracy reached 0.9572. The results demonstrate that the proposed sEMG-based recognition framework maintains high classification accuracy under real-time interactive conditions, suggesting the feasibility of the proposed framework for upper-limb nursing assistance scenarios.

## 4. Discussion

The comparative evaluation demonstrates that nonlinear ensemble-based classifiers outperform linear discriminant models in sEMG-driven motion intention recognition. Linear methods such as LDA and QDA exhibited limited performance, indicating that the selected time-domain feature space is not linearly separable across motion categories. In contrast, Random Forest and SVM achieved substantially higher accuracy, suggesting that nonlinear decision boundaries are better suited to capture the complex inter-class relationships inherent in multi-muscle activation patterns. The superior performance of Random Forest can be attributed to its ensemble structure, which reduces variance through bagging and improves robustness to local feature variations. Hyperparameter optimization further improved performance, confirming that model capacity plays a measurable role in classification stability under intra-subject evaluation settings. Beyond classification performance, this work emphasizes a system-level design that enables real-time and deployable sEMG-based intention recognition for interactive assistive applications. This distinguishes the proposed framework from existing studies that primarily focus on offline accuracy improvement using more complex models. In addition, prior studies have shown that both recognition latency and prediction stability significantly influence user perception and trust in human–robot interaction. The low-latency design and temporal consistency constraint adopted in this work are therefore expected to contribute not only to technical performance but also to improved interaction reliability from a user-centered perspective.

The feature evaluation results indicate that amplitude-related descriptors (RMS, IF, CF) provide stronger discriminative capability than statistical distribution measures such as variance or higher-order moments alone. RMS reflects effective muscle activation intensity, while impulse and clearance factors emphasize peak-related characteristics, which are closely associated with voluntary contraction strength. The selected triplet feature set achieved the highest overall performance, suggesting that combining global amplitude information with peak-sensitive metrics enhances class separability without introducing redundant dimensions. Nevertheless, minor confusion was observed between drinking and shoulder abduction motions during online validation. This phenomenon may be explained by partially overlapping muscle recruitment patterns during the early phase of movement execution. Similar activation amplitudes reduce inter-class distance within the selected feature space. This limitation indicates that time-domain features alone may not fully capture subtle inter-class differences; therefore, future work will explore temporal dynamics, frequency-domain features, and additional muscle channels to further enhance motion discriminability.

However, several limitations remain. In the current study, data acquisition was conducted under controlled conditions without active robotic assistance, and thus vibration-induced interference from mechanical actuation was not explicitly modeled. In practical applications, sEMG signals may be affected by electrode displacement, muscle fatigue, and motion artifacts. While these factors were mitigated under controlled experimental conditions, explicit robustness to such variations and long-term stability remain open challenges for real-world deployment. The current evaluation follows an intra-subject training paradigm, and cross-subject generalization was not investigated, which limits the adaptability of the framework across different users. This limitation highlights the importance of developing subject-independent models for reliable deployment in practical assistive scenarios. Additionally, only a limited set of four motion categories was evaluated under controlled laboratory conditions, which does not fully capture the complexity and variability of real-world activities of daily living. Expanding the motion repertoire and validating performance in more dynamic and less constrained environments will be essential for broader deployment in clinical or home-based rehabilitation settings. These directions will be critical for translating the proposed framework from controlled experimental settings to practical assistive applications.

## 5. Conclusions

This study presented a structured sEMG-based motion intention recognition framework for interactive upper limb nursing assistance. A standardized acquisition protocol and preprocessing pipeline were developed to ensure stable multi-channel signal recording under controlled conditions. Eight time-domain features were systematically evaluated, and a performance-oriented fusion strategy identified a compact feature triplet consisting of RMS, impulse factor, and clearance factor. Five supervised learning models were comparatively assessed, with Random Forest achieving the highest recognition performance. Further hyperparameter optimization using K-fold cross-validation led to measurable improvement in classification stability across subjects. The optimized model maintained consistent accuracy in a preliminary real-time online validation experiment. The findings demonstrate that computationally efficient time-domain feature fusion combined with ensemble-based classification can provide effective motion decoding for sEMG-driven interactive assistance systems. By preserving a clear separation between physiological signal interpretation and mechanical execution, the proposed framework ensures interpretable and stable system behavior. Although validation was conducted with a limited motion set under controlled laboratory conditions, the framework establishes a scalable foundation for expanding sEMG-based intention recognition in intelligent upper limb assistance applications.

## Figures and Tables

**Table 1:** The performance results of a single classifier for different actions under each feature.

	*y*_1_	*y*_2_	*y*_3_	*y*_4_	Mean Value
MAV	0.953	0.912	0.924	0.932	0.930
VAR	0.683	0.382	0.442	0.513	0.505
SK	0.946	0.882	0.911	0.881	0.905
KU	0.911	0.91	0.892	0.94	0.913
WF	0.922	0.924	0.874	0.92	0.910
IF	0.964	0.952	0.944	0.91	0.935
CF	0.976	0.938	0.896	0.937	0.937
RMS	0.969	0.936	0.914	0.937	0.939

**Table 2:** Performance results of single classifier for different actions under different feature fusion.

	*y*_1_	*y*_2_	*y*_3_	*y*_4_	Mean Value
RMS + MAV	0.963	0.921	0.946	0.94	0.942
RMS + VAR	0.781	0.715	0.72	0.746	0.74
RMS + KU	0.951	0.915	0.912	0.931	0.927
RMS + WF	0.941	0.937	0.914	0.925	0.929
RMS + IF	0.948	0.935	0.926	0.926	0.933
RMS + SK	0.964	0.911	0.912	0.91	0.924
RMS + MAV + CF	0.973	0.927	0.934	0.938	0.943
MAV + IF + CF	0.962	0.945	0.927	0.942	0.944
MAV + IF + RMS	0.951	0.943	0.946	0.943	0.945
IF + CF + RMS	0.964	0.945	0.936	0.943	0.947
MAV + IF + CF + RMS	0.97	0.92	0.93	0.92	0.941

**Table 3:** Action recognition accuracy based on Kf-Random Forest algorithm.

	*y*_1_	*y*_2_	*y*_3_	*y*_4_	Mean Value
Subject 1	0.9649	0.9543	0.9437	0.9529	0.9539
Subject 2	0.9627	0.9594	0.9585	0.9564	0.9592
Subject 3	0.9656	0.9552	0.9548	0.9481	0.9559
Subject 4	0.9592	0.9552	0.957	0.9507	0.9555
Subject 5	0.9522	0.9501	0.9593	0.9553	0.9542
Subject 6	0.9568	0.9446	0.9376	0.949	0.947
Subject 7	0.9638	0.9398	0.9488	0.9335	0.9464
Subject 8	0.9552	0.9459	0.9404	0.9428	0.9460
Average Accuracy	0.96005	0.950563	0.950013	0.948588	0.9523

## Data Availability

The data presented in this study are available upon reasonable request from the corresponding author.
